# Supplementary material for: Identification of photoperiod-induced specific miRNAs in the adrenal glands of Sunite sheep (Ovis aries)
Source: Front Vet Sci. 2022 Jul 22;9:888207. doi: 10.3389/fvets.2022.888207 (PMC9354845; doi:10.3389/fvets.2022.888207)
Supplement: Supplementary file 1 [file Data_Sheet_1.ZIP › Supplementary file/miRDeep2 file.zip!/miRDeep2 file/Know_Structure/SP42A1/oar-mir-329a.pdf]

miRBase precursor : oar-mir-329a
Total read count : 40
oar-miR-329a-5p read count : 3
oar-miR-329a-3p read count : 36
remaining reads : 1

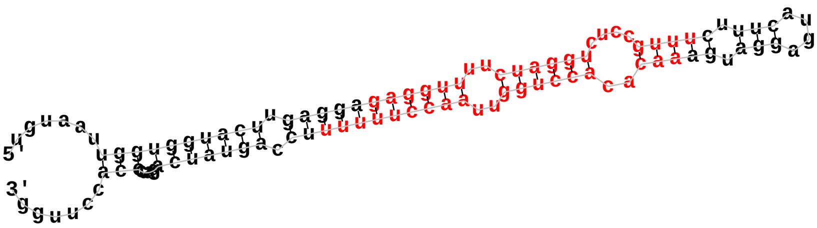

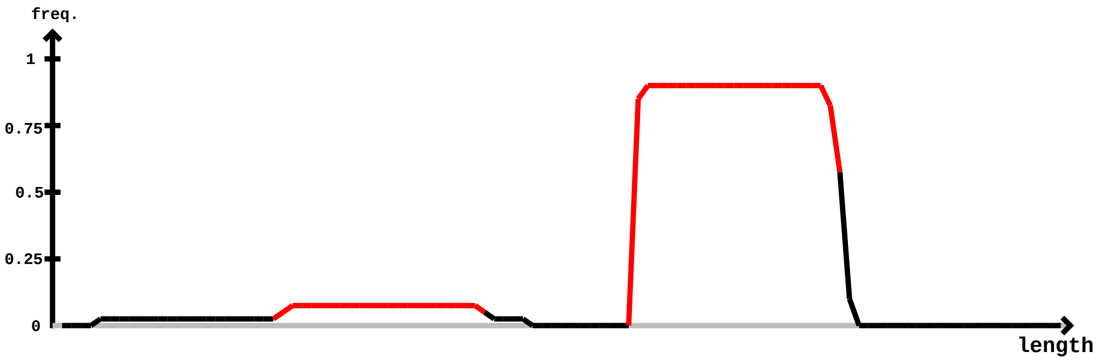


```
                                   oar-miR-329a-5p
                                                                   oar-miR-329a-3p
5'- uguaauuggugguacuugaggagagguuuucuaggucuccguuucuuucaugaggaugaaacacaccugguuaaccuuuuuuccaguaucagaucccaccuugg   -3'    exp
    .......(((((((((.(((((((((((..((((((....(((((.(((....))).)))))..))))))..))))))))))).)))).......)))))....    reads    mm    sample
    ...aauuggugguacuugagga..................................................................................    1    0    seq
    ......................gagguuuucuaggucuccguu.............................................................    1    0    seq
    ......................gagguuuucuaggucuccguuu............................................................    1    0    seq
    .......................agguuuucuaggucuccguuucuuu........................................................    1    0    seq
    ...........................................................aacacaccugguuaaccuuu.........................    3    0    seq
    ...........................................................aacacaccugguuaaccuuuu........................    10    0    seq
    ...........................................................aacacaccugguuaaccuuuuu.......................    18    0    seq
    ...........................................................aacacaccugguuaaccuuuuuu......................    3    0    seq
    ............................................................acacaccugguuaaccuuuuu.......................    1    0    seq
    ............................................................acacaccugguuaaccuuuuuu......................    1    0    seq
```
